# Comparison of Antibiotic Resistance and Virulence Factors among *Escherichia coli* Isolated from Conventional and Free-Range Poultry

**DOI:** 10.1155/2015/618752

**Published:** 2015-10-22

**Authors:** Vanessa L. Koga, Sara Scandorieiro, Eliana C. Vespero, Alexandre Oba, Benito G. de Brito, Kelly C. T. de Brito, Gerson Nakazato, Renata K. T. Kobayashi

**Affiliations:** ^1^Department of Microbiology, Laboratory of Basic and Applied Bacteriology, State University of Londrina (UEL), Rodovia Celso Garcia Cid, Caixa Postal 6001, 86051-980 Londrina, PR, Brazil; ^2^Department of Pathology and Clinical and Toxicological Analysis, State University of Londrina (UEL), Avenida Robert Koch, No. 60, Vila Operária, 86038-350 Londrina, PR, Brazil; ^3^Department of Zootechnia, State University of Londrina (UEL), Rodovia Celso Garcia Cid, Caixa Postal 6001, 86051-980 Londrina, PR, Brazil; ^4^Laboratory of Bird Health, Fepagro Animal Health, Veterinary Research Institute Desidério Finamor (IPVDF), Estrada do Conde, No. 6000, 92990-000 Eldorado do Sul, RS, Brazil

## Abstract

Microbiological contamination in commercial poultry production has caused concerns for human health because of both the presence of pathogenic microorganisms and the increase in antimicrobial resistance in bacterial strains that can cause treatment failure of human infections. The aim of our study was to analyze the profile of antimicrobial resistance and virulence factors of *E. coli* isolates from chicken carcasses obtained from different farming systems (conventional and free-range poultry). A total of 156 *E. coli* strains were isolated and characterized for genes encoding virulence factors described in extraintestinal pathogenic *E. coli* (ExPEC). Antimicrobial susceptibility testing was performed for 15 antimicrobials, and strains were confirmed as extended spectrum of *β*-lactamases- (ESBLs-) producing *E. coli* by phenotypic and genotypic tests. The results indicated that strains from free-range poultry have fewer virulence factors than strains from conventional poultry. Strains from conventionally raised chickens had a higher frequency of antimicrobial resistance for all antibiotics tested and also exhibited genes encoding ESBL and AmpC, unlike free-range poultry isolates, which did not. Group 2 CTX-M and CIT were the most prevalent ESBL and AmpC genes, respectively. The farming systems of poultries can be related with the frequency of virulence factors and resistance to antimicrobials in bacteria.

## 1. Introduction

Resistance to antimicrobial agents has become a major concern both for human health and in veterinary medicine. Antimicrobial agents are being used in many countries in veterinary practice for therapy and prophylaxis of infectious diseases and for growth promotion in food animals. However, the indiscriminate use of antimicrobials can result in bacterial selection pressure of the intestinal microbiota of animals [1–3]. Because multiresistant bacteria are frequently found in poultry meat [4–6], chicken products are suspected to be a source of foodborne pathogen and/or antimicrobial resistance bacteria for humans [1–3, 7, 8].

*Escherichia coli *have an important role within resistant bacteria populations, being widely used as a bioindicator of antimicrobial resistance and being pathogenic to humans and animals. Extraintestinal pathogenic* Escherichia coli* (ExPEC) can cause many human infections, such as septicemia, meningitis, and urinary tract infections, and can also cause disease in birds, being responsible for significant economic losses in poultry industry [1, 9]. ExPECs are characterized by the possession of many virulence factors including adhesins, toxins, iron acquisition systems, and serum resistance factors and, in phylogenetic classification, belong mainly to group B2 and occasionally to group D, whereas commensal* E. coli* belong to groups B1 and A [10, 11].

*β*-lactamase production is the most common mechanism of resistance for *β*-lactam in Gram-negative bacteria and is increasing in occurrence in humans, becoming a major public health problem [9]. However, *β*-lactamases of community and environmental origin have been discovered, for example, in food animals. Poultry are recognized as important carriers of *β*-lactamase-producing* E. coli*, and extended-spectrum *β*-lactamase (ESBL)/AmpC-producing bacteria in birds have been reported in many countries [12–14].

ESBL production confers resistance to 3rd- and 4th-generation cephalosporins but not to cephamycins (cefoxitin) and carbapenems and is inactivated by clavulanic acid. The AmpC enzymes confer resistance to 3rd-generation cephalosporins and cephamycins but are inhibited by *β*-lactamase inhibitors. Plasmid-mediated *β*-lactamases can carry multiple resistance genes non-*β*-lactaman, and their indiscriminate use can lead to coselection and/or coresistance in bacteria populations [9, 13, 15].

Many studies reported that there is a genetic similarity among avian and human ExPEC, leading to the hypothesis that meat animals play a role as reservoirs for drug-resistant bacteria and pathogenic bacteria [1, 16]. Little is known regarding the microbiological quality of chicken meat from different systems of poultry farming and their potential antimicrobial resistance and/or pathogenic behavior upon consumption. The aim of this study was to analyze the profile of virulence factors and antimicrobial resistance, including searching for ESBL/AmpC groups genes, in strains of* E. coli* isolated from conventional and free-range poultry carcass.

## 2. Material and Methods

### 2.1. Bacterial Isolates

A total of 156* E. coli* strains were isolated from commercial refrigerated chicken carcass, intended only for local consumption, sold in the city of Londrina (north region in Paraná, Brazil). Of these, 35* E. coli* strains were isolated from 15 free-range poultry (commonly created by family agriculture) and 121* E. coli* strains from 26 conventionally raised poultry (sold in markets in the region, obtained from granges) [17]. Each chicken carcass was placed into the sterile packaging with 100 mL of Brain Heart Infusion (Himedia Laboratories Pvt. Ltd., Mumbai, India). After homogenization, 0.1 mL was smeared onto MacConkey agar (Neogen Corporation Lansing, Michigan) and crystal violet red neutron bile agar (Neogen Corporation Lansing, Michigan) by pour plate. Both were incubated at 37°C for 18–24 h. Colonies suspected to be* E. coli *were confirmed by biochemical tests such as EPM, MILi [18, 19], and Simons citrate agar (Merck, KGaA, Darmstadt, Germany). One-to-eight strains were collected from each chicken carcass. Only strains that showed different genotypic characteristics of virulence factors and phenotypic resistance were selected.

### 2.2. Phylogenetic Classification

*E. coli* strains were assigned to phylogenetic groups (A, B1, B2, or D), according to the method of Clermont and collaborates [10]. This method is based on analysis of presence of the* chu*A and* yja*A genes and the DNA fragment (TSPE4.C2), as determined by Polymerase Chain Reaction (PCR). This PCR reaction contained 1.25 U Taq DNA polymerase (Life technologies, Rockville, MD) in 1x PCR buffer (Life technologies, Rockville, MD), 0.2 mM of each dNTP, 2.5 mM MgCl_2_, and 1 *μ*M of each primer. The conditions of PCR consisted of 94°C for 5 min followed by 30 cycles of 94°C for 30 s, 55°C for 30 s, and 72°C for 30 s with a final extension step at 72°C for 7 min. PCR amplicons were visualized on 2.0% agarose gels stained with GelRed (Biotium, Hayward, CA, USA). After gel electrophoresis, the images were captured using Image Capture Systems (LPixImageHE).

### 2.3. Virulence Factor Genes

Several virulence factors normally studied in ExPEC strains were surveyed. The selected genes were as follows:* iut*A (aerobactin siderophore receptor gene),* hly*F (putative avian hemolysin),* iss* (episomal increased serum survival gene),* iro*N (salmochelin siderophore receptor gene), and* omp*T (episomal outer membrane protease gene) [11]. This PCR contained 1.25 U Taq DNA polymerase (Life technologies, Rockville, MD) in 1x PCR buffer (Life technologies, Rockville, MD), 0.2 mM of each dNTP, 2.5 mM MgCl_2_, and 1 *μ*M of each primer. The conditions of PCR consisted of 94°C for 2 min, followed by 25 cycles of 94°C for 30 s, 63°C for 30 s, and 68°C for 3 min, with a final extension step at 72°C for 10 min [11]. PCR amplicons were visualized on 2.0% agarose gels stained with GelRed (Biotium, Hayward, CA, USA). After gel electrophoresis, the images were captured using Image Capture Systems (LPixImageHE).

### 2.4. Antimicrobial Susceptibility Testing

Antimicrobial susceptibility was performed using the standard disk diffusion method recommended by the Clinical and Laboratory Standards Institute [20, 21]. Antimicrobials used included 5 *μ*g of ciprofloxacin, 10 *μ*g of each of ampicillin, gentamicin, norfloxacin, and enrofloxacin, 30 *μ*g of each of cefazolin, cefotaxime, cefoxitin, ceftazidime, tetracycline, nalidixic acid, and chloramphenicol, 300 *μ*g of nitrofurantoin, 1.25/23.75 *μ*g of trimethoprim-sulfamethoxazole, and 20/10 *μ*g of amoxicillin-clavulanic acid (Oxoid Ltd., Basingstoke, Hants, UK). Strains resistant to third-generation cephalosporins were confirmed for ESBL production by double-disk diffusion testing between amoxicillin/clavulanate and cefotaxime or ceftazidime [22], or by using a combination disc test including cefotaxime, cefotaxime + clavulanic acid (Becton Dickinson, Sparks, MD), ceftazidime, and ceftazidime + clavulanic acid (Becton Dickinson, Sparks, MD), according to the CLSI recommendations. The strains positive in the phenotypic tests to ESBL production were screened to ESBL genes. Strains that showed cefoxitin and/or to 3rd-generation cephalosporins intermediate or resistance were tested by molecular screening of AmpC type genes. The* E. coli* isolate ATCC 25922 was used as a quality control to antimicrobial susceptibility testing, and the results were interpreted as per CLSI criteria.

### 2.5. Characterization of *β*-Lactamase Genes of ESBL and AmpC Groups

ESBL-producing* E. coli* was characterized for ESBL genes encoding CTX-M (1, 2, 8, 9, and 25 groups), TEM, and SHV type by PCR [23–25]. All isolates suspected by phenotypic tests for the production of AmpC were tested by a multiplex PCR described by Pérez-Pérez and Hanson [26]. Six family-specific plasmid mediated AmpC genes (MOX, FOX, EBC, ACC, DHA, and CIT) were evaluated. PCR amplicons were visualized on 2.0% agarose gels stained with GelRed (Biotium, Hayward, CA, USA). After gel electrophoresis, the images were captured using Image Capture Systems (LPixImageHE).

### 2.6. Statistical Analysis

Comparisons of frequencies among different groups were made by Fisher's exact test and Chi-square test. Findings were considered to be significant where *p* < 0.05. The test was performed with the statistical program R version 3.1.0.

## 3. Results

According to phylogenetic classification, the most prevalent group in strains from free-range poultry was the group A (54.3%), whereas the strains from conventionally raised poultry most frequently belonged to group B1 (37.2%), although no statistically significant differences were observed between them and groups B1, B2, and D (Table 1).

Regarding the search for virulence factors, we found significant difference for the majority of the genes studied between strains from free-range and conventional poultry, with the exception of the* iss* gene (*p* > 0.05) (Table 1). Few strains from free-range poultry were positive for virulence factors, with only 10 strains (28.6%) having at least one of the virulence factors studied. In contrast, 91 strains (75.2%) from conventionally raised poultry had at least one virulence factor.

**Figure 1:**
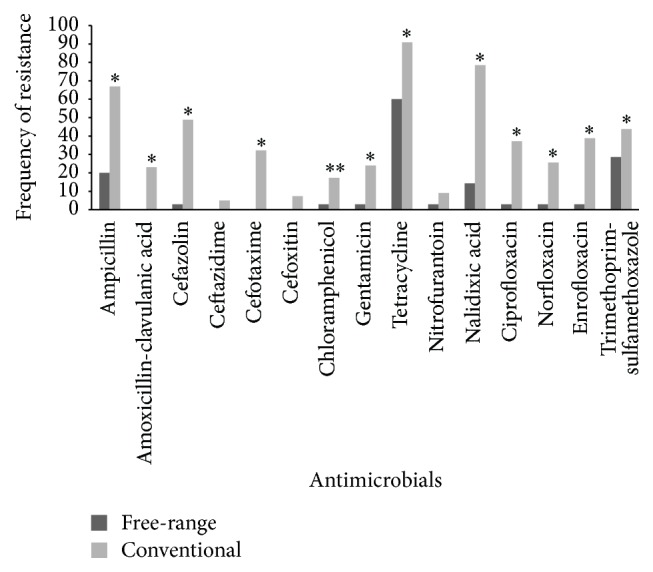
Frequency of antimicrobial resistance to* E. coli* strains isolated from free-range and conventional chicken carcass. ^*∗*^ *p* < 0.05, Chi-square test. ^*∗∗*^ *p* < 0.05, Fisher exact test.

According to the antimicrobial susceptibility test, strains from conventionally raised poultry showed a higher frequency of antimicrobial resistance than strains from free-range poultry for all antimicrobials tested (Figure 1). The frequency of antimicrobial resistance to strains from free-range poultry was low, except to tetracycline (60% of resistance), whereas the strains from conventional poultry showed a high frequency of resistance mainly to tetracycline, nalidixic acid, and ampicillin (Figure 1).

ESBL/AmpC genes appeared only in strains isolated from conventional poultry (42.1% of 121 strains from conventional poultry). Forty strains were ESBL-producing* E. coli*. The most prevalent group within these ESBL was the group 2 CTX-M (62.5% of ESBL-producing strains). Eleven strains showed only the CIT group of AmpC genes (9.1% of 121 strains from conventional poultry). No strain had ESBL and AmpC genes together (Table 2).

All ESBL/AmpC-producing strains showed resistance to one or more non-*β*-lactam antimicrobials, with resistance to tetracycline (98%) the most prevalent (Table 2).

We observed that ESBL/AmpC-producing strains were present in all four phylogenetic groups (A, B1, D, and B2), although there were few B2 strains. The majority of these strains were positive for at least one virulence factor.

## 4. Discussion

Many studies have demonstrated similarities between human and avian ExPEC, leading to the hypothesis that poultry products may serve as a source of ExPEC and are closely linked to human infections. Poultry meat exhibits the highest levels of* E. coli *contamination, and these are indicated as being more extensively antimicrobial-resistant than* E. coli* from other meats [27].

Avian* E. coli* often possess virulence genes similar to those found in human ExPEC [27]. We measured 5 virulence genes carried by plasmids that are normally studied in human ExPEC [28, 29] and used by Johnson and collaborates [11] to distinguish avian pathogenic avian* E. coli *(APEC) from commensal* E. coli*. Our results demonstrated that strains from conventionally raised poultry have a greater number of virulence genes than the strains from free-range poultry, with the exception of the* iss *gene (*p* > 0.05). Furthermore, few strains from free-range poultry showed virulence factors, unlike strains from conventionally raised poultry, of which 75.2% had at least one virulence factor. These genes were also found in* E. coli *isolated from urinary tract infections [30], and some of these genes (*iss*,* iro*N,* omp*T, and* hly*F genes) were found also in conjugative plasmid in human* E. coli* strains isolated from sepsis, in Brazil, indicating a possible zoonotic risks [28]. According to phylogenetic classification, our results showed most prevalence of group A in strains from free-range poultry and group B1 in strains from conventionally raised poultry. Thus, the majority of the strains show characteristics relative to commensal phylogenetic groups, although most strains from conventionally raised poultry were positive for virulence factors. These results can be related to the creation system because the conventional poultries are raised in larger groups in few areas, generating a high density, which facilitates the transmission of bacteria between them because there are many virulence genes carried by plasmids, whereas free-range poultry creation is in small groups, making it more difficult to transmit pathogens [13].

Antimicrobial resistance in bacteria isolated from food of animal origin is often associated with the use of antibiotics in livestock [2, 3, 8]. Due to indiscriminate use of antimicrobials in poultry feeds, since 2006, in Europe, the use of antimicrobials as growth promoters is prohibited [31]. The use of several antibiotics including tetracyclines, *β*-lactams, systemic sulfonamides, and quinolones has been banned as growth promoters in many countries, for example, in Brazil [32, 33].

In the antimicrobial susceptibility test, strains isolated from conventionally raised poultry showed a higher frequency of resistance than the strains from free-range poultry to all antimicrobials. There were significant differences for the majority of the antimicrobials tested, except for cefoxitin, ceftazidime, and nitrofurantoin (*p* > 0.05). The high frequency of antimicrobial resistance in strains from conventional poultry carcasses, primarily to tetracycline, nalidixic acid, and ampicillin, can be related with the selective pressure due to the high use of antimicrobials and/or the contamination of environment in aviculture industries.

However, an interesting finding in our study was the low frequency of antimicrobial resistance in strains from free-range poultry, except to tetracycline. It is known that the use of antimicrobials in family agriculture is restricted or even absent, being casually used for treating diseases [34]. Another hypothesis for the low observed frequency is that free-range poultry normally live in small groups, compared to conventionally raised poultry, leading to individual therapeutic interventions, whereas in the poultry industry, birds are kept in larger groups, so population-based therapeutics are mostly appropriate [13].

Tetracycline was the antimicrobial with the highest frequency of resistance in both rearing systems. The high frequency may be due to the easy access to and low price of these antimicrobials and poor monitoring by regulatory bodies in veterinary medicine in Brazil because these antimicrobials have prohibited use. Another explanation of the high frequency of resistance in strains from free-range poultry is its contact with environmental microorganisms, which produce natural antibiotics, or by soil contamination with the feces of wild animals that carry antibiotic-resistant microorganisms [8, 35].

*β*-lactam antimicrobials, especially the third-generation cephalosporins, are the most common treatment for human infections by Enterobacteriaceae. However, a large number of resistant bacteria have emerged worldwide. Among ExPEC, *β*-lactamases remain the most important mechanisms of *β*-lactam resistance. *β*-lactamases are hydrolytic enzymes that cleave the *β*-lactam ring. The emergence of *β*-lactamases is mainly linked to the spread of genes encoding ESBLs and/or plasmid-mediated AmpC *β*-lactamases [9]. However, ESBL/AmpC-producing bacteria are now being found in increasing numbers in food-producing animals, for example, in poultry meat [5, 13, 36].

One notable finding was the presence of ESBL/AmpC *β*-lactamases only in strains from conventional poultry, with group 2 CTX-M and CIT groups being the most prevalent ESBL and AmpC, respectively. The absence in strains from free-range poultry may indicate the low use of antimicrobials in its production.

CTX-M-type strains are the most common ESBL type in humans, despite several reports of TEM and SHV as well [9]. Other countries have also reported a high prevalence of ESBL-producing bacteria in poultry [5, 13, 36]. In Brazil, group 2 CTX-M has been identified in* Salmonella enteric *from chickens [37].

Plasmid-mediated AmpC genes are derived from chromosomal AmpC genes, the majority of plasmid-mediated AmpC genes being found in nosocomial isolates of* E. coli* and* Klebsiella pneumoniae*. Six families of plasmid-mediated AmpC *β*-lactamases have been identified [26]. Among AmpC, the CIT group was the most frequently observed in our results. Studies have related the presence of the CIT group in poultry in other countries [4, 12, 13]. In Brazil, the presence of plasmid-mediated AmpC-producing in human isolates has been sporadically reported [38, 39]. The presence of 11 AmpC-producing strains indicates the importance of studies both in human and in veterinary clinical practice.

Despite the increase of ESBL/AmpC-producing* E. coli* isolates in food-producing animals, little is known about the use of *β*-lactam because these are banned as growth promoters in Brazilian aviculture. One hypothesis is that the coselection and coresistance have taken place because the gene encoding ESBL and other classes of non-*β*-lactam can be located in the same mobile genetic element, such as plasmid or transposons [15]. In our study, ESBL/AmpC-producing strains showed resistance to one or more non-*β*-lactam antibiotics, mainly to tetracycline (98% of the cases). The presence of ESBL and AmpC gene was not observed in the same strain. It is possible that there is a limit to the amount of *β*-lactamase that a bacterial cell can accommodate and still be a viable pathogen [26].

We also note that the *β*-lactamases may be present in strains belonging to phylogenetic groups from commensal groups A and B1, as well as virulent strains from group D. We note also that the majority of ESBL/AmpC-producing strains have one or more virulence genes tested. This can indicate that some strains harbor antimicrobial resistance genes mediated by plasmids and perhaps are harboring virulence factors encoding genes mediated by other plasmids too. Some studies have shown that virulence plasmids and multidrug resistance plasmid were not found in the same strains [8, 40]. However, Johnson and collaborates [41] found in some APEC strains hybrid resistance plasmids encoding multiple resistance to both antimicrobials and virulence-associated genes that were able to infect human cells and cause meningitis in rats.

In our results, it is clear that even with the prohibition of many antimicrobials there is still a high frequency of antimicrobial resistance in strains from conventional poultry. The low frequency of antimicrobial resistance in strains from free-range poultry may indicate that the low use of antimicrobials in this system rearing may be related to the low frequency of resistance and virulence, which can lead to a low risk of transmission of pathogens or resistance genes to humans through consumption of chicken meat. The monitoring of antimicrobial resistance frequencies in animal foods can aid in the detection of banned poultry farming practices.

## 5. Conclusion

The high frequency of antimicrobial resistance, associated with several virulence factors, made* E. coli* in a potential food problem, due to the possibility of horizontal transfer of virulence genes and antimicrobial resistance to the human resident microbiota and/or human pathogens. The absence or restricted use of antimicrobials in free-range poultry production may be contributing to the lower frequency of bacterial virulence factors and resistance to antimicrobials, leading to a lower risk of their transmission to humans.

## Figures and Tables

**Table 1:** Prevalence of phylogenetic group and virulence genes in strains of *E. coli* isolated by free-range and conventionally raised poultry carcass.

	Free-range (*n* = 35)	Conventional (*n* = 121)
	Number of isolates (%)	Number of isolates (%)
Phylogenetic group		
A	19 (54.3)^*∗*^	35 (28.9)
B1	09 (25.7)	45 (37.2)
B2	00 (0)	5 (4.1)
D	07 (20)	36 (29.7)
Virulence genes		
* hlyF*	09 (25.7)	57 (47.1)^*∗*^
* iutA*	06 (17.1)	66 (54.5)^*∗*^
* iss*	07 (20)	43 (35.5)
* omp*T	08 (22.9)	64 (52.9)^*∗*^
* iro*N	01 (2.9)	35 (28.9)^*∗*^

^*∗*^ *p* < 0.05, Chi-square. Free-range versus conventionally raised poultry carcass *E. coli* isolates.

**Table 2:** Characteristics of *β*-lactamase genes and phenotypic antimicrobial resistance profile of strains of ESBL/AmpC-producing *E. coli*.

Isolate number	Phenotypic resistance profile	*β*-lactamase genes
1	Amp, amc, cfz, ctx, tet, nal	Group 1 CTX-M
2	Amp, kz, ctx, cn, tet, nal	Group 2 CTX-M
3	Amp, kz, ctx, cn, tet, nal, cip, nor, enr, sut	Group 2 CTX-M
4	Amp, kz, ctx, cn, tet, nal, cip, sut	Group 2 CTX-M
5	Amp, kz, ctx, cn, tet, nal, cip, enr	Group 2 CTX-M
6	Amp, kz, ctx, cn, tet, nal	Group 2 CTX-M
7	Amp, kz, ctx, clo, cn, nal, sut	Group 2 CTX-M
8	Amp, kz, ctx, tet, nal, cip, nor, enr, sut	Group 2 CTX-M
9	Amp, kz, ctx, tet, nal, cip, nor, enr, sut	Group 2 CTX-M
10	Amp, kz, ctx, clo, tet, nal	Group 2 CTX-M
11	Amp, kz, ctx, cn, tet, nal, cip, enr, sut	Group 2 CTX-M
12	Amp, kz, ctx, cn, tet, nal, enr	Group 2 CTX-M
13	Amp, kz, ctx, cn, tet, nal	Group 2 CTX-M
14	Amp, kz, ctx, cn, tet, nal, cip, sut	Group 2 CTX-M
15	Amp, amc, kz, cn, tet, nal, sut	Group 2 CTX-M
16	Amp, amc, kz, ctx, cn, tet, nal	Group 2 CTX-M
17	Amp, amc, kz, ctx, cn, tet, nal, cip, nor, enr, sut	Group 2 CTX-M
18	Amp, kz, ctx, cn, tet, nal	Group 2 CTX-M
19	Amp, kz, ctx, cn, tet, nal	Group 2 CTX-M
20	Amp, kz, ctx, clo, tet, nal, cip, nor, sut	Group 2 CTX-M
21	Amp, kz, ctx, cn, tet, nal, cip, enr	Group 2 CTX-M
22	Amp, amc, kz, ctx, tet, nit, nal	Group 2 CTX-M
23	Amp, amc, kz, ctx, cn, tet	Group 2 CTX-M
24	Amp, amc, kz, ctx, clo, tet, nit, nal, cip, nor, enr, sut	Group 8 CTX-M
25	Amp, kz, ctx, tet, enr	Group 8 CTX-M
26	Amp, kz, ctx, tet, nit	Group 8 CTX-M
27	Amp, amc, kz, ctx, clo, tet, nal, sut	Group 8 CTX-M
28	Amp, kz, ctx, tet	Group 8 CTX-M
29	Amp, kz, ctx, tet, nal, cip, nor, enr	Group 8 CTX-M
30	Amp, kz, ctx, tet	Group 8 CTX-M
31	Amp, kz, ctx, clo, tet, nal, cip, nor, enr	Group 8 CTX-M
32	Amp, kz, ctx, tet	Group 8 CTX-M
33	Amp, kz, ctx, caz, tet	Group 8 CTX-M
34	Amp, kz, ctx, tet, nit	Group 8 CTX-M
35	Amp, kz, ctx, tet, nal, cip, nor, enr	SHV
36	Amp, amc, kz, clo, cn, tet, nit, sut	CIT
37	Amp, amc, kz, cfo, tet, nal, sut	CIT
38	Amp, amc, kz, cfo, cn, tet, nal, sut	CIT
39	Amp, amc, kz, cfo, caz, tet, nal, sut	CIT
40	Amp, amc, kz, cfo, tet, nal, sut	CIT
41	Amp, amc, kz, cfo, caz, tet, nal, cip, nor, enr, sut	CIT
42	Amp, amc, kz, cfo, tet, nal, sut	CIT
43	Amp, amc, kz, cfo, clo, cn, tet, nal, cip, nor, enr, sut	CIT
44	Amp, amc, kz, cfo, ctx, tet, nal, sut	CIT
45	Amp, amc, kz, cfo, caz, tet, nal, cip, enr, sut	CIT
46	Amp, amc, kz, cfo, caz, tet, nit, nal, cip, enr	CIT
47	Amp, amc, kz, ctx, caz, clo, tet, nal, cip, nor, enr, sut	Group 1 CTX-M, Group 2 CTX-M
48	Amp, kz, ctx, tet, nal, cip, nor, enr, sut	Group 2 CTX-M, Group 8 CTX-M
49	Amp, amc, kz, ctx, tet, nal	Group 8 CTX-M, SHV
50	Amp, amc, kz, tet, nal	Group 8 CTX-M, SHV
51	Amp, amc, kz, ctx, clo, tet, nal, cip, nor, enr, sut	Group 2 CTX-M, Group 8 CTX-M, SHV

Ampicillin (AMP); amoxicillin-clavulanic acid (AMC); cefazolin (KZ); ceftazidime (CAZ); cefotaxime (CTX); chloramphenicol (CLO); gentamicin (CN); tetracycline (TET); nitrofurantoin (NIT); nalidixic acid (NAL); ciprofloxacin (CIP); norfloxacin (NOR); enrofloxacin (ENR); trimethoprim-sulfamethoxazole (SUT); not found (NF).
